# Reconstructive Alternative to the Osteocutaneous Free Flap in a Patient With Severe Peripheral Vascular Disease

**Published:** 2020-03-02

**Authors:** Nikhil R. Shah, Haripriya S. Ayyala, Radhika Malhotra, Soly Baredes, Ramazi O. Datiashvili

**Affiliations:** ^a^Division of Plastic Surgery, Department of Surgery; ^b^Division of Head and Neck Surgery, Department of Otolaryngology, Rutgers New Jersey Medical School, Newark

**Keywords:** free flap, head and neck cancer, mandible reconstruction, microsurgery, peripheral vascular disease

## CASE DESCRIPTION

A 54-year-old man received a diagnosis of squamous cell carcinoma of the floor of the mouth. The tumor extended to the symphyseal portion of the mandible and fungated through the skin in the submental area. After bilateral selective neck dissections and composite floor of the mouth, partial tongue, and mandible resections, the residual defect measured 168 cm^2^.

## QUESTIONS

1. What is the epidemiology of invasive head and neck cancers?
2. What are the goals of head and neck reconstruction?
3. What are common options for reconstruction of mandibular defects?
4. What impact does peripheral vascular disease have on flap selection?

## DISCUSSION

Globally, head and neck tumors comprise the sixth most common cancer, with squamous cell carcinoma representing nearly 90% of diagnoses.^1^ The most frequent causes are found to be alcohol and tobacco use, with a synergistic and dose-dependent effect in consumption and risk of disease.^2^ Recently, human papillomavirus (HPV) has also been shown to be a significant risk factor for the development of head and neck cancer, with an increased prevalence in the United States in the younger population.^3^

Invasive head and neck cancers are advanced in nature and often extend beyond the primary site to disrupt local anatomical integrity.^4^ The goals of reconstruction are to optimize function while maintaining a good aesthetic outcome. Plastic surgeons must consider versatile approaches to recreate both rigidity of the mandible and pliability of the oropharynx. From a functional standpoint, these structures are crucial for upper airway patency, mastication, and phonation.^5^ In addition, aesthetic restoration of the head and neck region should be ensured when possible. This area has significant implications for patients’ appearance and self-confidence.

For extensive mandibular defects, free tissue transfer is ideal to bring in healthy, vascularized tissue and for improved structural integrity. The most common options include the free fibular flap (FFF), iliac crest free flap, scapular or parascapular free flap, and radial forearm free flap. All of these flaps provide the opportunity to reconstruct both bony and cutaneous defects simultaneously. The FFF and the iliac crest flap offer large amounts of available bone as well as adequate vascular pedicles, allowing for variability in flap sizes. However, the peroneal vessels used in the FFF are commonly narrowed in patients with peripheral vascular disease and incidences of disfiguring scars and chronic gait disturbances following iliac crest flaps have been reported in as high as 20% of patients.^6^ The scapular flap is advantageous due to the general sparing of the circumflex scapular artery from atherosclerotic processes. The cutaneous paddle also tends to be comparable with facial skin color. However, this flap is preferred for reconstruction of lateral or angle defects and proves difficult to contour anteriorly.^6^^,^^7^ The radial forearm flap is predominantly used as a fasciocutaneous flap, though it can be prepared as an osteocutaneous option. However, the donor site commonly requires a split- or full-thickness skin graft for closure; delayed healing and wound breakdown have been reported in as high as 46% of patients and often result in underlying tendon exposure. These factors contribute to a high reoperation rate and an increased likelihood of chronic pain.^7^

**Figure 1:**
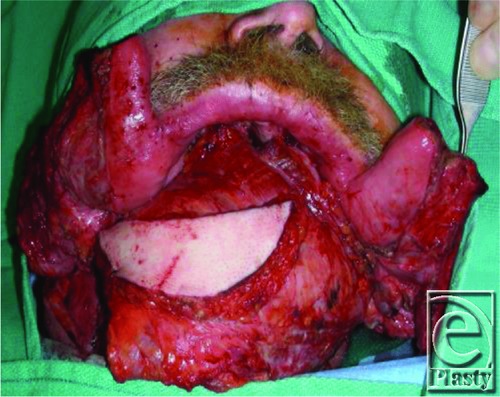
Latissimus dorsi myocutaneous free flap sandwiched around the titanium reconstructive plate.

**Figure 2:**
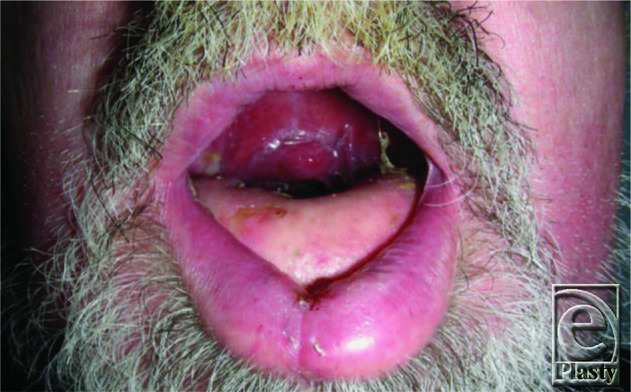
Postoperative outcome at 1-year follow-up.

Given the aforementioned causes of these cancers, a majority of patients expectedly reveal a long-standing history of peripheral vascular disease. In addition to causing technical difficulties with vascular anastomoses, it influences the choice of reconstruction. Our patient expressed an extensive history of smoking up to 4 packs per day; preoperative angiography revealed severely reduced diameter of the peroneal artery, precluding the possibility of using an FFF. The bony defect spanned the entire mandible, excluding the use of a scapular flap. Alternatively, the decision was made to perform bony reconstruction with a titanium plate. Next, recreation of soft-tissue bulk was achieved by sandwiching the plate between a latissimus dorsi myocutaneous free flap (Fig 1). This flap's diverse anastomotic blood supply provides for versatile flap harvesting while the caliber of the thoracodorsal system typically remains resistant to atherosclerotic damage. Furthermore, the bulk of the latissimus dorsi muscle ensures long-term flap durability and adequate coverage of any underlying hardware.^8^ The cutaneous paddle was subsequently utilized for closure of the oral floor and submental skin defect with good color matching. The superior thyroid artery and branch of the internal jugular vein were used as recipient vessels. The patient had an uncomplicated hospital course and was discharged on postoperative day 15. One year after the operation, functional and aesthetic results were satisfactory. Although the patient could not have dentures fitted because of lack of bony strut, he was able to tolerate a soft diet. The flap remained viable, and there was no evidence of exposed hardware or any underlying structures (Fig 2).
